# Clustering Algorithm Reveals Dopamine‐Motor Mismatch in Cognitively Preserved Parkinson's Disease

**DOI:** 10.1002/acn3.70317

**Published:** 2026-01-28

**Authors:** Rachele Malito, Chiara Meneghini, Alice Galli, Luca Gallo, Pierfrancesco Mitrotti, Paola Dimartino, Marianna Inglese, Cinzia Zatti, Alessandro Lupini, Andrea Pilotto, Alessandro Padovani, Micol Avenali, Enza Maria Valente, Cristina Tassorelli, Silvia P. Caminiti

**Affiliations:** ^1^ IRCCS Mondino Foundation Pavia Italy; ^2^ Department of Brain and Behavioral Sciences University of Pavia Pavia Italy; ^3^ Neurology Unit, Department of Clinical and Experimental Sciences University of Brescia Brescia Italy; ^4^ Department of Molecular Medicine University of Pavia Pavia Italy; ^5^ Department of Biomedicine and Prevention University of Rome Tor Vergata Rome Italy; ^6^ Department of Continuity of Care and Frailty, Neurology Unit ASST Spedali Civili of Brescia Brescia Italy

**Keywords:** biomarker, heterogeneity, neuroimaging, stratification

## Abstract

**Objective:**

To explore the relationship between dopaminergic denervation and motor impairment in two de novo Parkinson's disease (PD) cohorts.

**Methods:**

*n* = 249 PD patients from Parkinson's Progression Markers Initiative (PPMI) and *n* = 84 from an external clinical cohort. Clustering analysis stratified dopaminergic denervation, measured with ^123^I‐FP‐CIT‐SPECT, and motor impairment into mild [D and M] and severe [D+ and M+]. Differences in terms of biomarkers and clinical progression were assessed across subgroups. Causal mediation analysis evaluated the effect of co‐pathology on the relationship between subgroups and cognitive decline.

**Results:**

Four subgroups were identified. Two subgroups showed concordant profiles: the severe dopaminergic and motor impairment subgroup [D+/M+] exhibited poorer memory performance, pathological Aβ_1‐42_, as well as higher longitudinal Levodopa equivalent daily dose (LEDD) values and faster progression of motor disability; the mild dopaminergic and motor deficits [D/M] subgroup displayed a benign clinical profile and stable disease progression. Two subgroups exhibited dopaminergic and motor severity mismatch: the mild dopaminergic but severe motor impairment [D/M+] subgroup showed severe and rapidly progressive rigidity. CSF Aβ_1–42_ levels mediated the association between D+/M+ and cognitive decline in patients who were cognitively preserved at onset, accounting for 13% of the total effect. The external cohort supported the malignancy of D+/M+ and the presence of rigidity in D/M+.

**Interpretation:**

Concordant severe impairment reflects a malignant profile linked to Aβ‐related cognitive decline, while mild concordant cases show stable progression. Mismatch subgroups display distinct clinical patterns, underscoring the value of integrating imaging and motor features for early disease stratification.

## 1 Introduction

Striatal dopaminergic deficit is a supportive biomarker for PD diagnosis [1]. Dopamine transporter single‐photon emission computed tomography (DAT‐SPECT) is the main neuroimaging technique used in both clinical research and practice to assess the presence and the degree of nigrostriatal dopaminergic dysfunction [2].

However, the relationship between motor impairment and striatal DAT‐SPECT uptake is still unclear [3, 4], with recent findings showing a correlation between the degeneration of the less affected putamen and the progression of motor symptoms [5].

Here, capitalizing on findings suggesting the utility of DAT‐SPECT to monitor disease progression and tracking individual disease trajectory [5], we aim to assess the relationship between dopaminergic denervation and motor impairment in cognitively preserved de novo PD cohorts. Further, we aim to examine how the mismatch between DAT‐SPECT and motor impairment—using clinical and imaging measures available worldwide—relates to the underlying pathology and disease progression.

## 2 Methods

### 2.1 Participants

Data used in this study was acquired from the Parkinson's Progression Markers Initiative (PPMI) database between July 1, 2010, and May 1, 2016 (www.ppmi‐info.org/access‐data‐specimens/download‐data), RRID:SCR_006431. The datasets analyzed in the present study are not publicly available due to PPMI repository (www.ppmi‐info.org/access‐data‐specimens/download‐data). For up‐to‐date information on the study, visit www.ppmi‐info.org [6].

Our sample comprised 249 de novo PD patients (mean age ± SD: 62.95 ± 9.04 years; sex [F/M]: 83/166). All participants met the following inclusion criteria: (1) a clinical diagnosis of idiopathic PD based on the diagnostic criteria of the International Parkinson and Movement Disorders Society; (2) disease duration ≤ 2 years; (3) absence of prior treatment; and (4) availability of a ^123^I‐FP‐CIT SPECT scan to assess brain dopaminergic density. Patients with moderate or advanced PD (Hoehn and Yahr stage ≥ III), a diagnosis of dementia at baseline, or a longitudinal clinical follow‐up ≤ 1 year were excluded. Since longitudinal changes in Montreal Cognitive Assessment (MoCA) scores were the primary outcome of interest, patients with a baseline diagnosis of mild cognitive impairment (MCI) were excluded to ensure a homogeneous cohort at baseline and avoid bias related to the higher risk of cognitive decline associated with this condition [7, 8].

Patients underwent an average of 8 years of clinical follow‐up (mean ± SD: 7.71; range: 1–13 years), during which comprehensive demographic and clinical assessments were conducted.

In full compliance with the Declaration of Helsinki principles, each PPMI participating site received approval from their local ethics committee and written informed consent was obtained from all participants.

### 2.2 Clinical and Neuropsychological Assessment

All PPMI subjects underwent a comprehensive clinical motor and non‐motor assessment.

The motor evaluation included the MDS‐UPDRS part II and III which respectively assess the motor difficulties in daily life and the global motor burden [9]. Based on MDS‐UPDRS part III, we divided the severity of motor impairment into tremor (sum of items 15–18), rigidity (item 3), bradykinesia (sum of items 2, 4–9 and 14) and postural (sum of items 1 and 9–13) [10].

At baseline, all patients were drug‐naïve and therefore assessed in the OFF state. During follow‐up, MDS‐UPDRS part III scores were available either in the ON or OFF state. To account for treatment exposure, each clinical evaluation was systematically paired with the corresponding levodopa equivalent daily dose (LEDD, ±3 months tolerance) which was inserted as a nuisance variable. LEDD was defined as the cumulative exposure of patients to all dopaminergic drugs. We also included the presence of deep brain stimulation (DBS) advanced therapy as a variable of interest.

The non‐motor assessment included the MDS‐UPDRS part I, which evaluates the non‐motor aspects of daily living experiences, the Scales for Outcomes in PD‐Autonomic (SCOPA‐AUT) [11], which assesses autonomic dysfunction, the rapid eye movement (REM) sleep behavior disorder‐screening questionnaire (RBDSQ) that verifies the presence of sleep disturbances [12], with RBD disorders defined as a score ≥ 6 [13]. The neuropsychiatric evaluation included the Geriatric Depression Scale (GDS) [14], Questionnaire for Impulsive‐Compulsive Disorders in Parkinson's Disease (QUIP‐Current‐Short) (QUIPcs) [15], and anxiety inventory (STAI) subscales: STAI state (STAI‐S) and STAI trait (STAI‐T) [16].

Global cognition was assessed through MoCA [17]. Other considered cognitive tests included the Benton Judgment of Line Orientation (JOLO) [18], the Hopkins Verbal Learning Test (HVLT) [19] symbol digit modalities test (SDM) [20], letter number sequencing (LNS) [21], and the semantic fluency test [22].

The longitudinal changes in the MoCA were calculated by determining individual slopes for each participant (MoCA slope) [23]. These slopes were derived using linear regression models ‐ adjusted for age, sex, and education ‐ considering MoCA scores as the dependent variable and time as the independent variable.

### 2.3 DAT‐SPECT Imaging Analysis

All participants underwent DAT‐SPECT; reconstructed images are available on the PPMI site (www.ppmi‐info.org/data). Images preprocessing was conducted with Statistical Parametric Mapping (SPM12). Each reconstructed image passed a quality check before being included. After images' spatial normalization (http://www.nitrc.org/projects/spmtemplates) [24], specific binding ratio (SBR) was obtained considering lateral superior occipital cortex as reference [25].

ROIs were selected from the Automated Anatomical Labelling (AAL) atlas [26]. The posterior fornix was considered the boundary between anterior and posterior putamen. SBR in the posterior putamen was used to calculate the asymmetry index (AI) through Walker et al. formula [27].

DAT‐SPECT SBR data from both cohorts were harmonized across acquisition sites using the NeuroCombat (empirical‐Bayes ComBat for neuroimaging) algorithm (Python v3.6, neuroCombat package) [28], adjusting explicitly for site‐specific batch effects (Model Machine) in nonparametric empirical Bayes mode.

### 2.4 CSF Biomarkers

Baseline data for α‐syn seeding aggregation activity (SAA) (one sample per participant) were downloaded from the PPMI biospecimen database. Samples were categorized as positive for α‐syn SAA if all three replicates were positive, negative if zero or one replicate showed positivity, or inconclusive if two replicates were positive. The Aprion α‐syn SAA assay, developed by Concha‐Marambio et al. [29] is described elsewhere in full details [30].

Data on the levels of α‐syn [31], Aβ_1‐42_, total Tau (t‐Tau), phosphorylated Tau (p‐Tau_181_) [32], and neurofilament light chain (NfL) in the CSF were also considered. Ratios between α‐syn, Aβ_1‐42_, t‐Tau, and p‐Tau_181_ CSF biomarkers were calculated following the guidelines outlined by Kang et al. [32].

### 2.5 External Cohort

To assess whether the agreement between motor and dopaminergic classifications would show comparable baseline and follow‐up characteristics, we performed a clustering analysis on an external cohort.

Inclusion and exclusion criteria are the same used for the PPMI cohort. The dataset included 84 participants who were enrolled at Brescia Hospital in Italy (mean age ± SD: 64.55 ± 9.4 years; sex [M/F]: 52/32). Participants underwent baseline clinical evaluations—including MDS‐UPDRS part III, presence/absence of cardinal motor symptoms, LEDD, RBDSQ, and Mini‐Mental State Examination (MMSE)—and DAT‐SPECT acquisitions, together with a longitudinal follow‐up (mean duration: 5.27 ± 2.4 years) of MDS‐UPDRS part III and LEDD.

DAT‐SPECT acquisition at baseline was performed 3 h after tracer administration using a Discovery 630 scanner (General Electric, Milwaukee, WI). Images were reconstructed via filtered back projection, with a three‐dimensional Butterworth post‐filter (order 10.0; cutoff 0.50 cycles/cm) and corrected for attenuation using Chang's method (attenuation coefficient 0.15 cM1). The same pre‐processing pipeline applied for the PPMI cohort was adopted.

The external cohort was included as part of a study protocol approved by the Ethics Committee of Brescia Hospital, Brescia, Italy (DNA study, NP 1471). Written informed consent was obtained from each participant. The study was conducted in accordance with the Declaration of Helsinki. Data are available upon reasonable request.

### 2.6 Statistical Analysis

**FIGURE 1:**
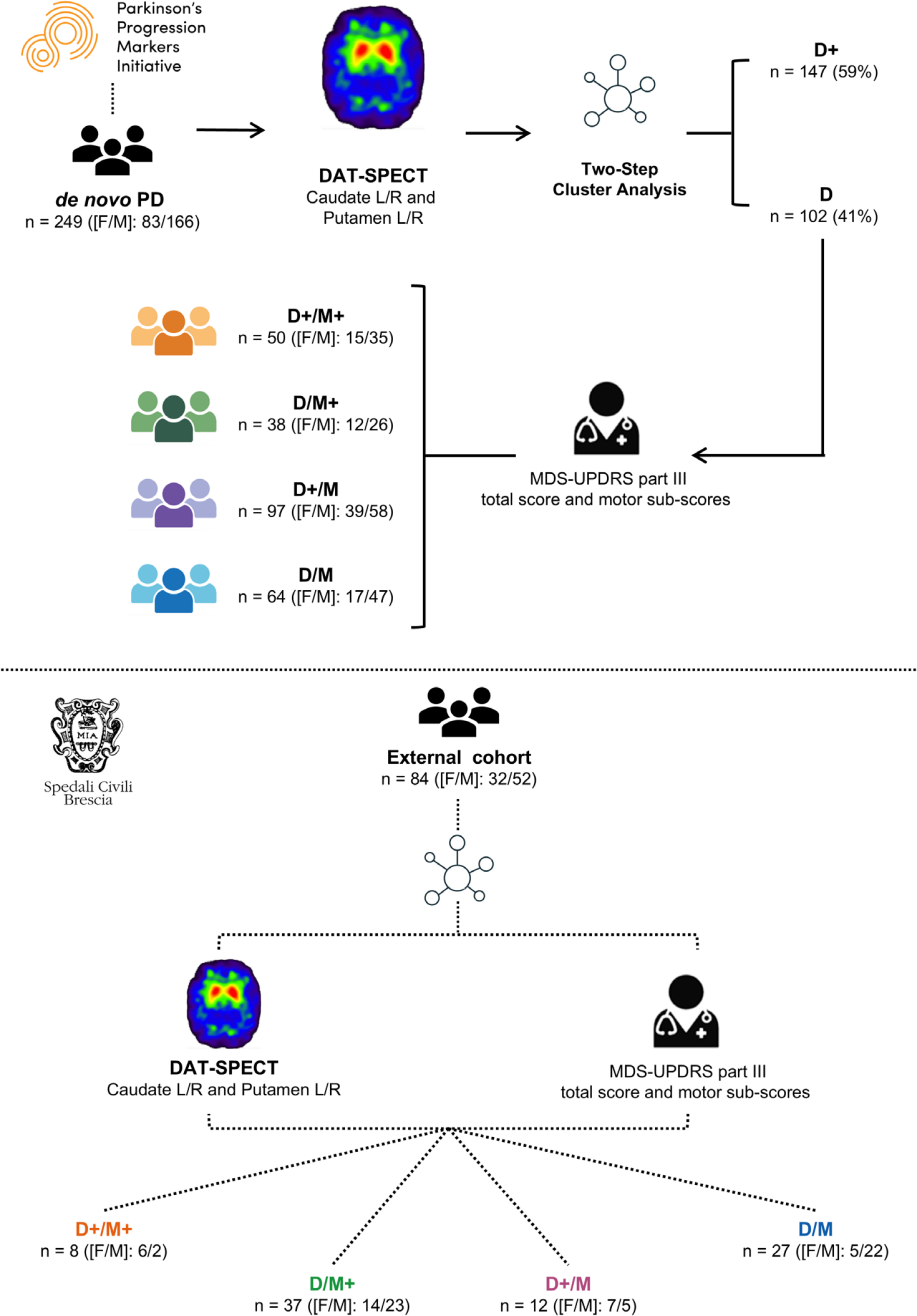
Methodological framework of cluster analysis. Classification of a de novo Parkinson's disease (PD) cohort (*n* = 249) using DAT‐SPECT imaging and MDS‐UPDRS‐III, followed by Two‐Step Cluster Analysis to define distinct subgroups. The same procedure was performed on an external cohort (*n* = 84). DAT, dopamine active transporter; L, left; MDS‐UPDRS, Movement Disorders Society—Unified Parkinson's Disease Rating Scale; PD, Parkinson's disease; R, right; SPECT, single photon emission computerized tomography.

To explore empirical clusters within the PD cohort we ran two independent two‐step cluster analyses, which combined a pre‐clustering stage with an agglomerative hierarchical procedure and used the log‐likelihood distance measure. The first analysis considered only harmonized DAT‐SPECT SBR extracted from the caudate and putamen regions (*Dopamine features*, “D”); the second clustering was performed considering MDS‐UPDRS III total score and motor sub‐scores: bradykinesia, postural, rigidity, and tremor (*Motor feature*, “M”). For both clustering analyses the algorithm was configured to autonomously determine the best number of clusters based on the Bayesian Information Criterion and requiring a silhouette measure ≥ 0.5. Standardization and outlier handling (noise threshold set at 25%) were managed within the SPSS preprocessing pipeline. No missing data were detected among the clustering predictors. For each clustering solution we saved the obtained classification of PD patients (“D”: 0 = mild; 1 = severe; “M”: 0 = mild; 1 = severe). Finally, we combined the two independent binary classifications to obtain the final subgroups (D+/M+ = 1/1; D/M = 0/0; D/M+ = 0/1; D+/M = 1/0) (Figure 1). To further evaluate cluster quality, we computed cluster‐level mean silhouette values and the proportion of negative silhouettes using Python (scikit‐learn, silhouette_samples function), based on the cluster assignments generated by the two‐step procedure.

Chi‐squared and one‐way ANOVA were performed to compare demographic features of clusters and subgroups. Moreover, patients were classified based on a previously proposed classification [33], into “mild motor‐predominant,” “intermediate,” and “diffuse malignant.”

Clinical, CSF, and *DAT‐SPECT* characteristics (dependent variables) were evaluated using linear and logistic regression models considering clusters and subgroups as independent variables. Analyses were performed adjusting for sex, age, and disease duration at baseline. Multiple comparisons were adjusted using the Benjamini‐Hochberg (BH) test applied globally across all pairwise comparisons. Specifically, the BH‐adjusted *p* values were computed using the *p*.adjust function in R with method = “BH”, which ranks the *p* values and adjusts them based on their rank and the total number of tests to control the false discovery rate. Variables that were used to generate the clusters were excluded from the set of tests considered in the multiple comparison correction.

The longitudinal rate of change in *motor* outcomes—including LEDD, MDS‐UPDRS part III sub‐scores for bradykinesia, tremor, rigidity, postural instability—was analyzed using a linear mixed‐effects (LME) model. This model was adjusted for age, sex, education level, DBS and LEDD (excluding DBS and LEDD in the treatment model) and accounted for repeated measurements within individuals by including a random intercept to capture interindividual variability in motor scores and a random slope to model individual trajectories over time. Time was modeled as a continuous variable, defined as the difference in years between the baseline assessment and each subsequent visit. The LME method was chosen for its robustness in handling correlated data within subjects, as well as its ability to manage missing data, irregular follow‐up intervals, and unbalanced group sizes [34]. LME was also performed to assess disease progression rates across the four subgroups. In this analysis, each subgroup was compared both to the overall sample and in pairwise comparisons between subgroups.

We applied a causal mediation analysis framework (R mediation package) to investigate whether the association between D+/M+ and longitudinal changes in MoCA scores (MoCA slope) was mediated by CSF biomarkers.

To analyze the causal model, we fitted two linear regression models, that is, the mediator and outcome models. Specifically, the mediator model considered the CSF biomarker as the dependent variable and D+/M+ as the independent variable, and the outcome model considered MoCA slope as the dependent variable and both D+/M+ and the CSF biomarker as the independent variables. Each model was adjusted for age, sex, and education. We estimated the total effect, the indirect effect (average causal mediation effect, ACME), and the direct effect (average direct effect, ADE) for each model. Nonparametric bootstrapping (1000 replications) was used to generate quasi‐Bayesian 95% confidence intervals for the estimated effects [35, 36]. Causal mediation was considered significant when the ACME showed a *p* < 0.05. The proportion of the total effect mediated by each CSF biomarker was expressed as a percentage.

*Sensitivity analysis on the external cohort*—The same clustering, clinical comparisons (using nonparametric Kruskal–Wallis and Chi‐square tests), and longitudinal analyses were applied independently to the external cohort, replicating the pipeline used for the PPMI cohort (see Supplementary Methods for further details).

Two‐tailed *p* values were considered for all statistical analyses.

All statistical analyses were performed using R software, version 3.6.3.

## 3 Results

### 3.1 Cluster Analysis

*DAT‐SPECT* classification identified two clusters: D+ (severe denervation) and D (mild denervation) with a sample size of 147 (59%) and 102 (41%), respectively (Silhouette = 0.6).

The motor classification provided two clusters: M+ (severe deficit) and M cluster (milder deficit), with a sample size of 88 (35.3%) and 161 (64.7%), respectively (Silhouette = 0.5). Detailed clustering results and silhouette statistics are shown in Tables S1, S2, and Figure S1.

**FIGURE 2:**
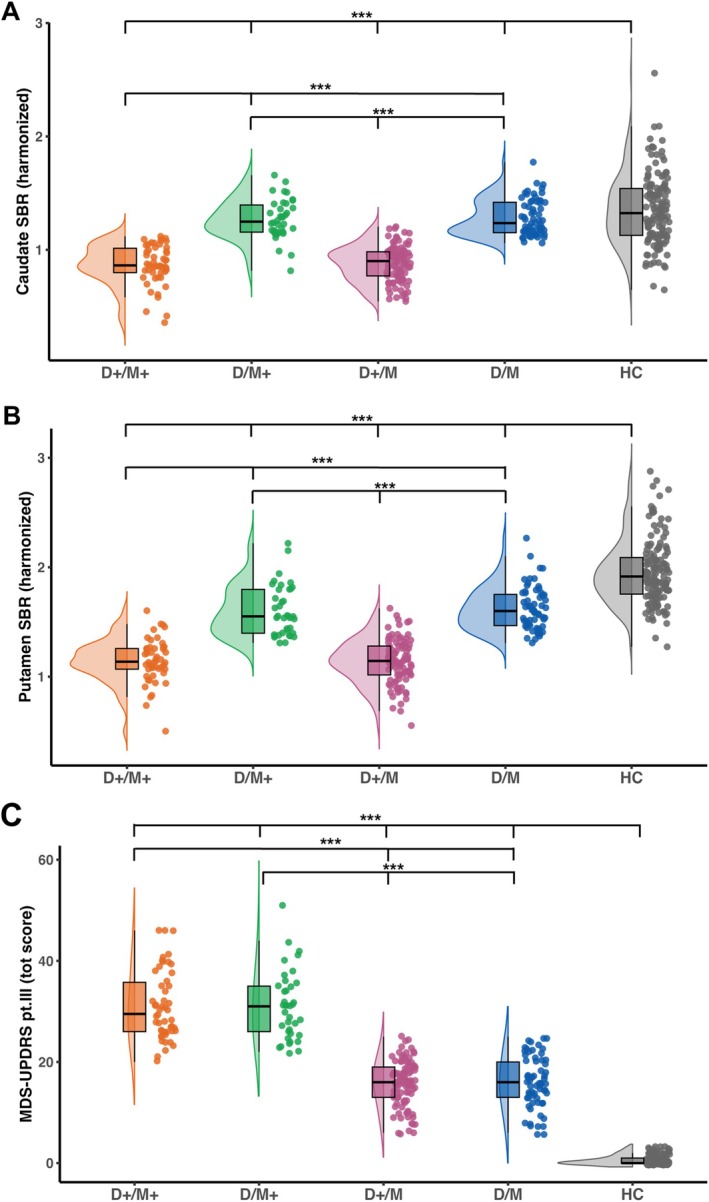
Clustering solution of PPMI cohort. Identification of four subgroups using Two‐Steps Clustering analysis based on harmonized (A) caudate and (B) putamen *DAT*‐*SPECT* uptake, and (C) MDS‐UPDRS III total score. DAT, dopamine active transporter; HC, healthy controls; MDS‐UPDRS, Movement Disorders Society—Unified Parkinson's Disease Rating Scale; SBR, specific binding ratio; SPECT, single photon emission computerized tomography. Significance: *p ≤ 0.05; **p ≤ 0.01; ***p ≤ 0.001. Subgroups differ significantly from HC (*p* < 0.001).

By combining the severity of motor and dopaminergic impairment, four subgroups emerged (Table S3). All subgroups exhibited lower uptake in the caudate and putamen and MDS‐UPDRS part III total score compared to HC (Figure 2).

### 3.2 Differences in Baseline and Follow‐Up Data Between Dopamine Clusters

D+/D clusters were similar for age, age at onset, sex, education, and disease duration (Table S4). D+ exhibited lower memory performances than D cluster. See Tables S4–S6 for further details.

At follow‐up, D displayed a nonsignificant trend toward greater tremor than D+ cluster (0.75 point) (Table S7).

### 3.3 Differences in Baseline and Follow‐Up Data Among Subgroups

The four subgroups were similar for age, age at onset, sex, education, and disease duration (Table S8).

**FIGURE 3:**
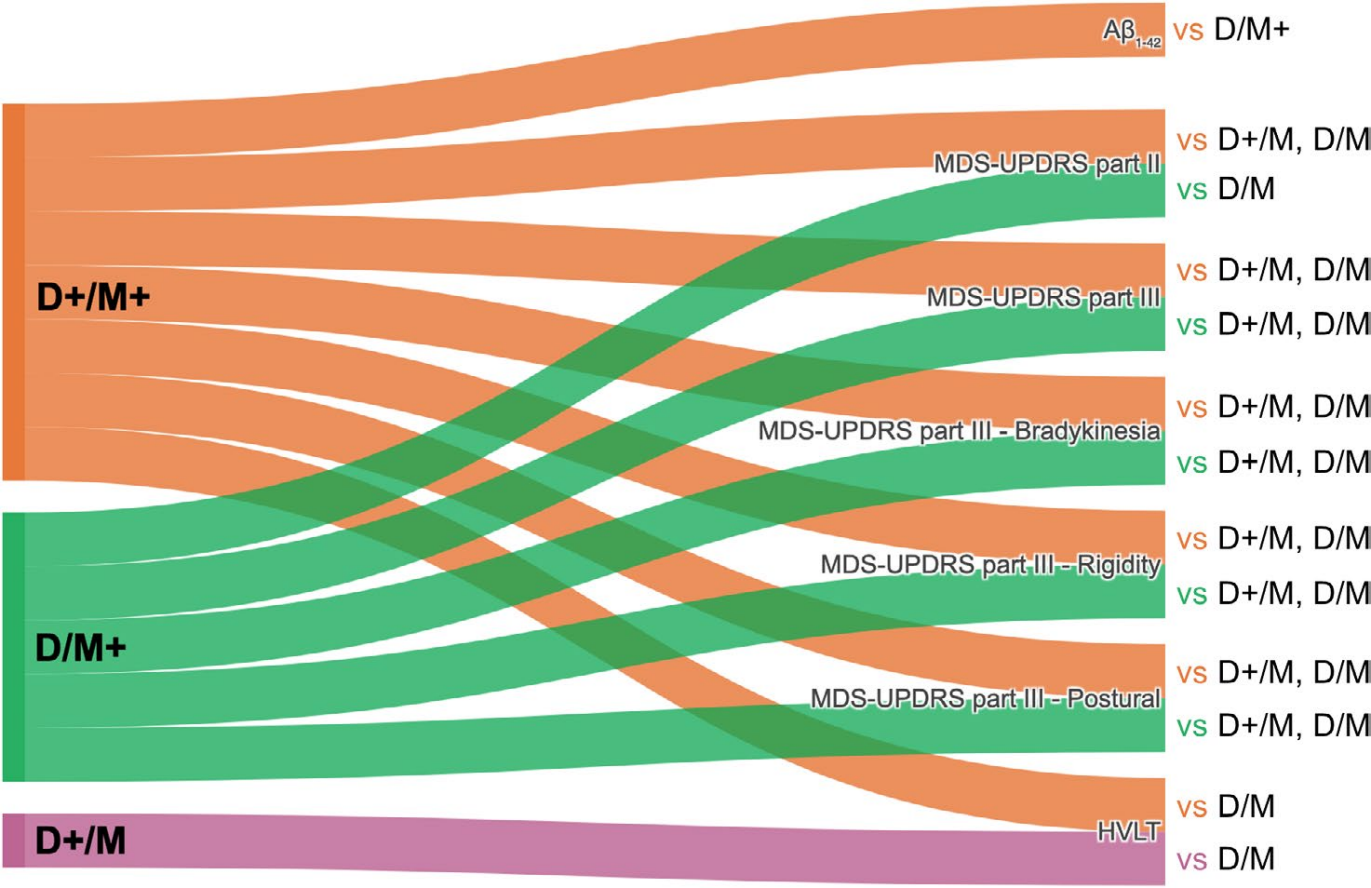
Sankey Diagram representing associations between clinical and biological variables across Parkinson's Disease subgroups in the PPMI cohort. Representation of the associations between clinical and biological variables and the four Parkinson's disease subgroups. Lines represent variables that remained significant after Benjamini–Hochberg correction (*p* < 0.05). On the right, direct comparisons that survived Benjamini–Hochberg correction are reported. Amyloid‐β_1‐42_, Aβ_1‐42_; HVLT, Hopkins Verbal Learning Test; MDS‐UPDRS, Movement Disorders Society—Unified Parkinson's Disease Rating Scale.

At baseline, both D+/M+ and D/M+ exhibited more severe MDS‐UPDRS part III, bradykinesia, postural instability, rigidity and more severe MDS‐UPDRS part II than M subgroups. D+/M+ and D+/M exhibited lower memory performance than D/M. Additionally, D+/M+ showed more pathological levels of Aβ_1‐42_ than D/M+ (Tables 1, 2, 3 and Figure 3). D/M revealed the most benign presentation of the disease. Consistently, 71.9% of D/M subjects were classified as “mild motor‐predominant phenotype” (Table S3).

**TABLE 1:** Comparison of baseline clinical features among the four subgroups of Parkinson's disease of the PPMI cohort.

	D+/M+	D/M+	D+/M	D/M	*p*					
					D+/M+ vs. D/M+	D+/M+ vs. D+/M	D+/M+ vs. D/M	D/M+ vs. D+/M	D/M+ vs. D/M	D+/M vs. D/M
*N* (%)	50 (20.1%)	38 (15.3%)	97 (39%)	64 (25.7%)	—	—	—	—	—	—
Clinical assessment										
MDS‐UPDRS I	1.5 (2.2)	1.1 (1.4)	1.4 (1.7)	1.0 (1.3)	0.296	0.649	0.206	0.537	0.875	0.208
Hallucinations and psychosis	0.02 (0.1)	0.03 (0.2)	0.03 (0.2)	0.03 (0.2)	0.880	0.799	0.749	0.986	0.960	0.954
Apathy	0.22 (0.7)	0.34 (0.6)	0.22 (0.5)	0.19 (0.4)	0.393	0.875	0.821	0.172	0.134	0.732
Pain	0.76 (0.8)	0.82 (0.8)	0.79 (0.9)	0.58 (0.8)	0.691	0.883	0.239	0.468	0.132	0.269
Constipation	0.48 (0.6)	0.42 (0.6)	0.052 (0.8)	0.27 (0.4)	**0.024**	0.545	**0.038**	0.339	0.254	**0.016**
Fatigue	0.72 (0.8)	0.76 (0.8)	0.68 (0.8)	0.52 (0.7)	0.851	0.499	0.211	0.412	0.144	0.337
MDS‐UPDRS II	7.6 (4.0)	7.4 (4.5)	5.2 (3.4)	4.2 (2.9)	0.850	**< 0.001** ^**a**^	**< 0.001** ^**a**^	**0.004**	**< 0.001** ^**a**^	**0.045**
SCOPA‐AUT	10.9 (6.2)	10.3 (6.1)	11.1 (6.7)	9.2 (5.2)	0.615	0.493	0.138	0.151	0.428	**0.018**
Constipation	1.5 (1.5)	0.84 (0.9)	1.2 (1.4)	0.9 (1.1)	**0.024**	0.565	**0.024**	**0.025**	0.651	**0.035**
Orofacial	1.1 (1.2)	1.4 (1.5)	0.96 (1.1)	0.77 (1.0)	0.391	0.344	0.086	0.084	**0.015**	0.304
Cardiovascular	0.48 (0.8)	0.53 (1.1)	0.52 (0.7)	0.27 (0.5)	0.713	0.927	0.064	0.987	0.091	**0.015**
Thermoregulatory	1.0 (1.3)	1.0 (1.4)	1.3 (1.5)	0.9 (1.1)	0.997	0.447	0.620	0.484	0.738	0.164
Urinary	4.2 (3.1)	4.2 (2.6)	4.5 (3.1)	3.7 (2.7)	0.899	0.296	0.371	0.131	0.436	**0.033**
Sex	1.24 (1.8)	0.95 (1.3)	1.3 (1.6)	1.4 (1.8)	0.389	0.462	0.710	0.069	0.202	0.893
Pupillomotor	0.26 (0.4)	0.45 (0.7)	0.39 (0.6)	0.34 (0.6)	0.133	0.172	0.414	0.710	0.399	0.706
RBDsq	3.7 (3.0)	3.3 (2.8)	3.1 (2.8)	3.1 (2.4)	0.550	0.186	0.138	0.711	0.692	0.949
RBD disorders (RBDsq ≥ 6)	13 (26%)	10 (26.3%)	16 (16.5%)	8 (12.5%)	0.847	0.123	**0.048**	0.208	**0.040**	0.370
QUIPcs	3.3 (1.7)	3.8 (1.8)	3.8 (1.6)	3.4 (1.7)	0.245	**0.046**	0.757	0.964	0.261	0.091
GDS	5.3 (1.6)	4.9 (1.1)	5.3 (1.5)	5.2 (1.3)	0.138	0.486	0.582	0.246	0.247	0.879
STAI S	44.9 (10.7)	48.1 (3.8)	46.7 (6.5)	48.4 (5.2)	0.090	0.224	**0.029**	0.299	0.677	0.087
STAI T	45.6 (4.1)	46.5 (3.3)	46.1 (4.0)	46.0 (3.9)	0.323	0.655	0.525	0.540	0.629	0.983

*Note:* Linear and logistic regression models (with the Benjamini‐Hochberg correction) were performed to compare clinical features at baseline between subgroups. Missing value: MDS‐UPDRS II (*n* = 1); SCOPA‐AUT—RBD—STAI—GDS—QUIPcs (*n* = 3). Missing value: MDS‐UPDRS II (*n* = 1); SCOPA‐AUT—RBD—STAI—GDS—QUIPcs (*n* = 3). Significant *p* values (*p* < 0.05) are reported in bold.

Abbreviations: GDS, Geriatric Depression Scale; MDS‐UPDRS, Movement Disorder Society—Unified Parkinson's Disease Rating Scale; *N*, number of participants or observations in a study or subgroup; QUIPcs, Questionnaire for Impulsive‐Compulsive Disorders in Parkinson's Disease–Short Form; RBDsq, REM Sleep Behavior Disorder Screening Questionnaire; SCOPA‐AUT, Scales for Outcomes in Parkinson's Disease–Autonomic; STAI S, State–Trait Anxiety Inventory—State Subscale; STAI T, State–Trait Anxiety Inventory—Trait Subscale.

^a^ Significance survived Benjamini‐Hochberg's correction.

**TABLE 2:** Comparison of baseline cognitive features among the four subgroups of Parkinson's disease of the PPMI cohort.

	D+/M+	D/M+	D+/M	D/M	*p*					
					D+/M+ vs. D/M+	D+/M+ vs. D+/M	D+/M+ vs. D/M	D/M+ vs. D+/M	D/M+ vs. D/M	D+/M vs. D/M
*N* (%)	50 (20.1%)	38 (15.3%)	97 (39%)	64 (25.7%)	—	—	—	—	—	—
Cognitive assessment										
MoCA (at baseline)	26.9 (2.9)	26.4 (2.1)	26.9 (2.6)	27.3 (2.4)	0.434	0.467	0.357	0.631	0.050	0.105
MoCA Slope	−0.645 (1.1)	−0.09 (1.0)	−0.20 (0.7)	−0.19 (0.6)	**0.016**	**0.025**	**0.011**	0.206	0.413	0.527
Follow‐up time (years)	6.6 (3.4)	6.4 (3.8)	7.8 (3.4)	8.3 (3.0)	0.867	0.065	**0.005**	**0.041**	**0.005**	0.358
BJLO	12.5 (2.6)	12.2 (2.9)	13.3 (2.3)	13.5 (1.6)	0.687	**0.034**	**0.047**	**0.024**	**0.023**	0.736
LNS	11.9 (2.1)	12.0 (2.6)	11.4 (3.1)	11.8 (2.6)	0.871	0.170	0.785	0.176	0.513	0.255
Semantic fluency	50.8 (10)	50.8 (8.4)	49.8 (10.7)	51.8 (8.9)	0.964	0.479	0.645	0.495	0.596	0.215
HVLT, immediate recall	43.8 (8.8)	47.7 (10)	44.8 (11.8)	47.1 (10.3)	0.075	0.925	0.054	0.095	0.947	**0.049**
HVLT, delayed recall	42.4 (11)	45.1 (11.8)	43.8 (11.5)	47.0 (10)	0.329	0.795	**0.014**	0.391	0.316	**0.016**
HVLT, retention	45.4 (12.3)	45.4 (12.2)	46.8 (11.8)	49.3 (10)	0.935	0.770	**0.035**	0.706	**0.049**	0.091
HVLT, recognition index	39.2 (12)	45.4 (10.7)	44.3 (11.8)	48.8 (9.5)	**0.019**	0.059	**< 0.001** ^**a**^	0.378	0.055	**< 0.001** ^**a**^
SDM	43.8 (8.9)	44.8 (11)	44.5 (8.6)	46.9 (9.0)	0.707	0.717	0.066	0.902	0.245	**0.044**

*Note:* Linear and logistic regression models (with the Benjamini‐Hochberg post hoc correction) were performed to compare cognitive features at baseline between subgroups. Missing value: BJLO—LNS—Semantic fluency—HVLT—SDM (*n* = 4). Significant *p* values (*p* < 0.05) are reported in bold.

Abbreviations: Benton Judgment of Line Orientation (BJLO); Hopkins Verbal Learning Test‐Revised (HVLT); Letter‐Number Sequencing (LNS); Montreal Cognitive Assessment (MoCA); Number of participants or observations in a study or subgroup (*N*); Symbol Digit Modality (SDM).

^a^ Significance survived Benjamini‐Hochberg's correction.

**TABLE 3:** Comparison of baseline biomarker features among the four subgroups of Parkinson's disease of the PPMI cohort.

	D+/M+	D/M+	D+/M	D/M	*p*					
					D+/M+ vs. D/M+	D+/M+ vs. D+/M	D+/M+ vs. D/M	D/M+ vs. D+/M	D/M+ vs. D/M	D+/M vs. D/M
*N* (%)	50 (20.1%)	38 (15.3%)	97 (39%)	64 (25.7%)	—	—	—	—	—	—
Biomarkers										
SAA (*)					0.485					
Inconclusive	0 (0.0%)	2 (5.7%)	2 (2.1%)	0 (0.0%)	—	—	—	—	—	—
Positive	42 (93.3%)	31 (88.6%)	87 (92.6%)	58 (95.1%)	—	—	—	—	—	—
Negative	3 (6.7%)	2 (5.7%)	5 (5.3%)	3 (4.9%)	—	—	—	—	—	—
α‐syn	1365.4 (537.8)	1667.9 (719.9)	1540.4 (762.9)	1589 (719.1)	**0.045**	0.178	0.083	0.571	0.824	0.927
Aβ_1‐42_	747.2 (275.6)	1115.9 (599.1)	937.7 (463.2)	899.7 (357.7)	**< 0.001** ^**a**^	**0.015**	**0.018**	0.112	**0.036**	0.491
p‐Tau_181_	12.9 (4.4)	16.1 (6.7)	14.5 (5.0)	15.4 (5.4)	0.111	0.231	0.132	0.495	0.787	0.725
t‐Tau	157.3 (54.2)	186.3 (72.6)	165.6 (55.3)	177.1 (54.9)	0.057	0.287	0.067	0.214	0.665	0.435
NfL	15 (10.7)	15.5 (7.0)	13 (6.6)	10.7 (4.8)	0.901	0.685	0.107	0.749	**0.013**	**0.034**
p‐Tau_181_/α‐syn	0.01 (0.002)	0.01 (0.002)	0.009 (0.002)	0.01 (0.002)	0.620	0.539	0.259	0.295	0.571	**0.038**
t‐Tau/α‐syn	0.12 (0.02)	0.12 (0.02)	0.11 (0.02)	0.12 (0.03)	0.933	0.121	0.716	0.156	0.693	**0.025**
Aβ_1‐42_/α‐syn	0.59 (0.2)	0.69 (0.2)	0.62 (0.2)	0.61 (0.2)	**0.002**	0.130	0.298	0.060	0.064	0.889
p‐Tau_181_/Aβ_1‐42_	0.019 (0.007)	0.015 (0.007)	0.015 (0.004)	0.019 (0.01)	**0.036**	**0.015**	0.765	0.656	**0.042**	**0.024**
t‐Tau/Aβ_1‐42_	0.218 (0.08)	0.182 (0.07)	0.182 (0.05)	0.223 (0.1)	**0.016**	**0.005**	0.853	0.721	**0.026**	**0.011**
p‐Tau_181_/t‐Tau	0.084 (0.009)	0.084 (0.007)	0.084 (0.006)	0.086 (0.007)	0.848	0.803	0.287	0.583	0.265	0.329

*Note:* Linear and logistic regression models (with the Benjamini‐Hochberg post hoc correction) were performed to compare biomarker features at baseline between subgroups. Missing value: SAA (*n* = 14); α‐syn (*n* = 11); t‐Tau (*n* = 17); p‐Tau_181_ (*n* = 30); Aβ_1‐42_ (*n* = 14); NfL (*n* = 11). (*) Indicates variables analyzed using the chi‐square test. Significant *p* values (*p* < 0.05) are reported in bold.

Abbreviations: Aβ_1‐42_, amyloid‐β_1‐42_; *N*, number of participants or observations in a study or subgroup; NfL, neurofilament light chain; p‐Tau_181_, phosphorylated tau; SAA, α‐syn seeding aggregation activity; t‐Tau, total tau; α‐syn, α‐synuclein.

^a^ Significance survived Benjamini‐Hochberg's correction.

**FIGURE 4:**
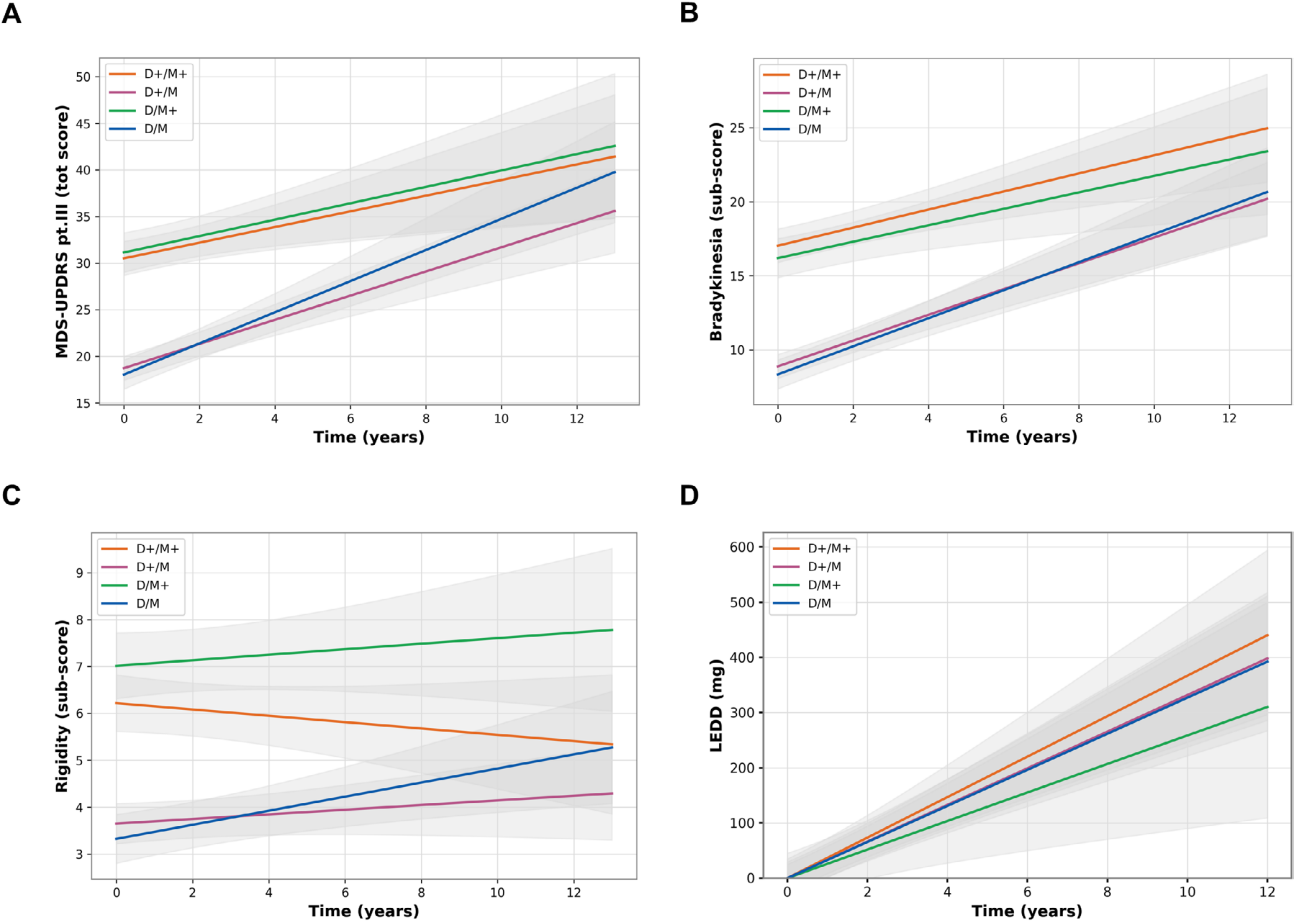
Temporal trends in motor scores across four Parkinson's Disease subgroups in the PPMI cohort. Clinical changes across time points in (A) Movement Disorders Society‐Unified Parkinson's Disease Rating Scale (MDS‐UPDRS) part III total score; (B) Bradykinesia sub‐score; (C) Rigidity sub‐score; (D) Levodopa equivalent daily dose (LEDD).

At follow‐up, D+/M+ and D/M+ showed a marked greater annual rate of change in MDS‐UPDRS part III motor scores than M subgroups. Specifically, the D+/M+ showed the most prominent changes in bradykinetic (7.02 points) and postural symptoms (0.91 points); conversely, the D/M+ showed more severe rigidity symptoms (2.87 points) than M subgroups. Of note, only the D+/M+ showed a significant increase of LEDD consumption over time (48.6 points) compared to all the other subgroups (Figure 4, Tables S9 and S10).

### 3.4 Mediation Analysis

Only Aβ_1‐42_ levels showed a significant indirect effect mediating the association between D+/M+ and longitudinal changes in MoCA slope. The average total effect of D+/M+ on MoCA slope (−0.38; 95% CI −0.744 to −0.088; *p* < 0.004) was decomposed into the ADE (−0.33, 95% CI −0.702 to −0.027; *p* = 0.020) and the ACME (−0.05; 95% CI −0.11 to −0.01; *p* = 0.008).

The proportion of the total effect of D+/M+ on MoCA slope causally mediated by Aβ_1‐42_ was 13.0% (95% CI 6.60–112.8; *p* = 0.012) (Table S11).

### 3.5 Sensitivity Analysis

In the external cohort, D+/M+ exhibited a higher prevalence of bradykinetic symptoms (100%), while D/M+ showed a predominance of rigidity‐related symptoms (71%). Longitudinally, both D+ and D+/M+ displayed a significantly faster progression of motor impairment (see Supplementary Results for further details, Table 4 and Table S12).

**TABLE 4:** Comparison of the baseline clinical features and longitudinal rate of change among the four subgroups of the external Parkinson's disease cohort.

	D+/M+	D/M+	D+/M	D/M	*p*
*N* (%)	8 (9.5%)	37 (44.1%)	12 (14.3%)	27 (32.1%)	—
Age at baseline	70.1 (5.2)	63.3 (10.3)	63.3 (9.6)	65.2 (8.8)	0.351
Age at onset	69.8 (5.1)	62.6 (10.4)	61.8 (9.4)	64.5 (8.7)	0.269
Sex (% of males)	2 (25%)	23 (62.2%)	5 (41.7%)	22 (81.5)	**0.010**
Education (years)	7.3 (4.5)	8.9 (4.2)	11.6 (3.2)	9.4 (3.6)	0.106
Disease Duration (years)	1.4 (0.5)	1.2 (0.4)	1.4 (0.5)	1.4 (0.5)	0.432
LEDD at baseline	0.00 (0.0)	0.00 (0.0)	0.00 (0.0)	0.00 (0.0)	—
Clinical assessment at baseline					
MDS‐UPDRS‐III	26.1 (18.6)	12.2 (6.8)	7.8 (3.4)	11.2 (7.4)	**0.018**
Bradykinesia	7/7 (100%)	24/31 (77.4%)	0/8 (0%)	0/24 (0%)	**< 0.001**
Rigidity	3/7 (42.9%)	22/31 (71%)	0/8 (0%)	0/24 (0%)	**< 0.001**
Resting tremor	3/7 (42.9%)	20/31 (64.5%)	8/8 (100%)	19/24 (79.2%)	0.055
RBD	0/3 (0%)	5/11 (45.5%)	3/4 (75%)	5/9 (55.6%)	0.338
MMSE	28.6 (1.8)	27.5 (1.5)	26.4 (3.6)	28.2 (1.7)	0.203

Clinical trajectories at follow‐up				
Rate of change in motor score				
Reference: Group	LEDD		MDS‐UPDRS‐III score	
	Estimate (95% CI)	*p*	Estimate (95% CI)	*p*
D+/M+	0.73 (−54.5 to 54.9)	0.979	14.8 (8.79 to 20.9)	**< 0.001**
D/M+	−2.33 (−35.6 to 30.9)	0.891	−1.27 (−5.76 to 3.22)	0.579
D+/M	−13.2 (−62.8 to 36.5)	0.604	−4.33 (−11.3 to 2.6)	0.221
D/M	9.98 (−25 to 45)	0.576	−3.27 (−8.04 to 1.49)	0.178

*Note:* Chi‐square and the nonparametric Kruskal–Wallis tests were used to compare baseline demographic and clinical features across subgroups. The longitudinal comparison between subgroups was performed using a linear mixed‐effects model. Significant *p* values (*p* < 0.05) are reported in bold.

Abbreviations: LEDD, Levodopa equivalent daily dose; MDS‐UPDRS, Movement Disorders Society—Unified Parkinson's Disease Rating Scale; MMSE, Mini‐Mental State Examination; *N*, number of participants or observations in a study or subgroup; RBD, REM sleep Behavior Disorder.

## 4 Discussion

This study demonstrates that early concordance or mismatch between dopaminergic dysfunction and motor impairment may play distinct roles in shaping the clinical phenotype of PD. Leveraging the rich longitudinal data of the PPMI cohort, we investigated the clinical manifestation and trajectories of disease progression in a cohort of de novo cognitively intact PD patients at baseline and we identified distinct patterns across subtypes. The key results were independently replicated in a consecutive external cohort of patients with PD.

To this aim, we used DAT‐SPECT imaging, a supportive biomarker for PD diagnosis [1], to systematically identify two distinct clusters (D/D+), reflecting milder and more severe dopaminergic damage, respectively. Importantly, imaging data were acquired at baseline, in the early clinical phase and in the absence of treatment and cognitive impairment, ensuring unbiased assessment of dopaminergic denervation. Furthermore, we investigated the characteristics of discordance between dopaminergic damage and motor impairment in terms of co‐pathology and clinical outcomes. Our findings revealed that dopaminergic and motor impairment can be discordant with clinical implications for disease progression.

Indeed, the subgroups identified at baseline, despite having similar disease duration, age at onset and SAA positivity, showed different clinical and biomarker features and rates of PD progression. The recent development of SAAs techniques allowed for the in vivo detection and measurement of misfolded α‐syn [37, 38]. However, as also suggested by our findings, in PD, SAA positivity per se is not useful for disease phenotypic stratification.

By focusing on dopaminergic degeneration, we highlighted that the D+ cluster was associated with greater memory impairment. Our results are consistent with previous evidence showing that cognitive impairment in PD typically emerges in the context of significant dopaminergic denervation, particularly affecting the caudate nucleus [39].

When combining dopaminergic and motor impairment, we identified four subgroups.

In detail, D+/M+ exhibited the most severe disease phenotype, marked by pronounced motor dysfunction—bradykinesia and postural instability—alongside memory deficits. These observations confirmed previous evidence proposing both severe motor dysfunction and dopaminergic denervation at baseline as the cause of a more malignant PD phenotype [33]. We also found a causal mediation effect of Aβ_1‐42_ on the association between D+/M+ and cognitive decline. The literature has extensively reported the predictive value of Aβ_1‐42_ for cognitive decline in PD [40]. Moreover, recent studies have shown that Aβ_1‐42_ has a synergistic role with tau, α‐synuclein and neurodegeneration thereby contributing to the complex pathological interactions that shape PD progression [41, 42, 43]. These findings indicate that single‐mediator models, while informative, capture only part of this biological interplay, and that future studies should incorporate multiple biomarkers to better characterize these interacting pathways. In line with this, differences in CSF Aβ_1‐42_ may also reflect clinical characteristics that are not fully matched across subgroups, indicating that residual confounding may contribute to the observed associations and should be considered when interpreting this finding [44].

At follow‐up, the severity of initial motor symptoms and dopaminergic loss not only predicts faster progression of motor impairment—but also correlates with an increased dependency on dopaminergic therapies over time. This subgroup is therefore expected to require more intensive healthcare resources. The severity of this subgroup was validated in the external cohort of patients which confirmed the prominent and quickly progressive motor impairment.

Conversely, the D/M group exhibited a relatively mild clinical profile, with the most favorable long‐term prognosis and the highest proportion of patients classified as “mild motor‐predominant phenotype.” In line with this, recent evidence has shown that individuals with the mild motor‐predominant phenotype experience progressive atrophy in the basal ganglia, likely reflecting ongoing dopaminergic loss during the early disease stage. By contrast, in the diffuse‐malignant subtype, most dopaminergic neurons appear to be already severely compromised in the initial years, as supported by more pronounced baseline abnormalities in DAT scans, suggesting a saturation effect [45].

The remaining two subgroups [D/M+ and D+/M] demonstrated a clear mismatch between dopaminergic denervation and motor phenotype, further highlighting the complex and multifactorial nature of PD pathology. In detail, the D/M+ subgroup exhibited a relatively more compromised motor profile, specifically characterized by heightened rigidity. Rigidity has recently been studied through biomechanical and neurophysiological measures, suggesting a partial overlap between the neural circuits involved in long‐latency reflexes and those contributing to “objective rigidity” in PD [46]. It has been proposed that the spino‐cerebello‐reticulo‐spinal pathway may drive parkinsonian rigidity, potentially explaining the observed severity of motor impairment even in cases of relatively mild striatal dopaminergic damage [46].

The D+/M subgroup exhibited more severe dopaminergic denervation despite a milder motor phenotype. This apparent discrepancy may reflect individual differences in motor reserve, which can mitigate the clinical impact of substantial dopaminergic loss. Indeed, previous studies have shown that factors such as education, premorbid physical activity, and the side of symptom onset (dominant vs. nondominant) can shape motor reserve, thereby influencing a patient's capacity to compensate for PD‐related pathology [47, 48, 49]. Importantly, both the D/M+ and D+/M subgroups showed higher NfL levels compared with the D/M subgroup, indicating greater overall burden of neuroaxonal damage that, in the D/M+ subgroup, does not primarily target nor exclusively involve the dopaminergic system. Consistently, elevated NfL levels have been associated with widespread neurodegeneration across cortical, subcortical, and brainstem regions in PD [50]. In addition, recent evidence indicates that higher NfL levels correlate with motor severity and are associated with an increased risk of both motor and cognitive progression in PD [51].

In the external cohort, we confirmed two key findings: D+/M+ showed both a higher prevalence of bradykinetic symptoms at baseline and a significantly faster progression of motor impairment over time, consistent with its malignant profile, while D/M+ reproduced the rigidity‐dominant profile. However, the limited biomarker and clinical data available prevented us from testing the full spectrum of subgroup features observed in the PPMI cohort.

Due to the nonconsecutive recruitment strategy adopted by PPMI, we cannot ascertain that the considered cohort of patients is fully representative of the general PD population, particularly regarding the potential underrepresentation of patients with more severe phenotypes who may be more prone to dropout. Also, the exclusion of individuals with MCI at baseline may have limited the generalizability of findings and reduced the ability to capture the full spectrum of motor–dopamine dissociation and its clinical relevance. Future studies in larger and more diverse cohorts, including patients with MCI, will be important to validate these findings across the full disease spectrum and ensure broad applicability of the proposed phenotypes across clinical settings. Another limitation concerns the use of LEDD, which does not adequately reflect interindividual variability in levodopa responsiveness [52]. As a result, some residual variability in ON/OFF motor states may not have been fully captured by the LME model. Future studies could benefit from integrating direct measures of levodopa responsiveness, such as ON/OFF UPDRS‐III scores, to enhance the precision of motor outcome modeling.

In addition, the future adoption of clustering approaches that integrate non‐motor and extra‐dopaminergic features, with greater granularity in subgroup definition, could provide further insights into the heterogeneity and progression of PD.

Despite these limitations, our findings contribute to understand how the concordance or mismatch between dopaminergic derangement and motor presentation may help stratify PD from its early clinical stages.

## Author Contributions

S.P.C. were responsible for the study design and conceptualization; R.M., C.M., A.G., M.I., and P.D. contributed to the analyses and the interpretation of data. S.P.C., R.M., and C.M. drafted the manuscript and prepared the figures. L.G., P.M., C.Z., and A.L. processed data and revised the manuscript. A.Pi., A.Pa., M.A., E.M.V., and C.T. contributed to data interpretation. All authors reviewed and approved the final manuscript.

## Funding

The authors have nothing to report.

## Conflicts of Interest

The authors declare no conflicts of interest.

## Supporting information


**Figure S1:** Visualization of group separation using Linear Discriminant Analysis (LDA).

**Appendix S1:** acn370317‐sup‐0002‐Appendix.docx.

## Data Availability

The PPMI datasets analyzed in the present study are not publicly available due to the PPMI repository. The external cohort dataset is available upon reasonable request.
