# Supplementary figures and images for: Clustering Algorithm Reveals Dopamine‐Motor Mismatch in Cognitively Preserved Parkinson's Disease
Source: Ann Clin Transl Neurol. 2026 Jan 28;13(7):1398–411. doi: 10.1002/acn3.70317 (PMC13358561; doi:10.1002/acn3.70317)

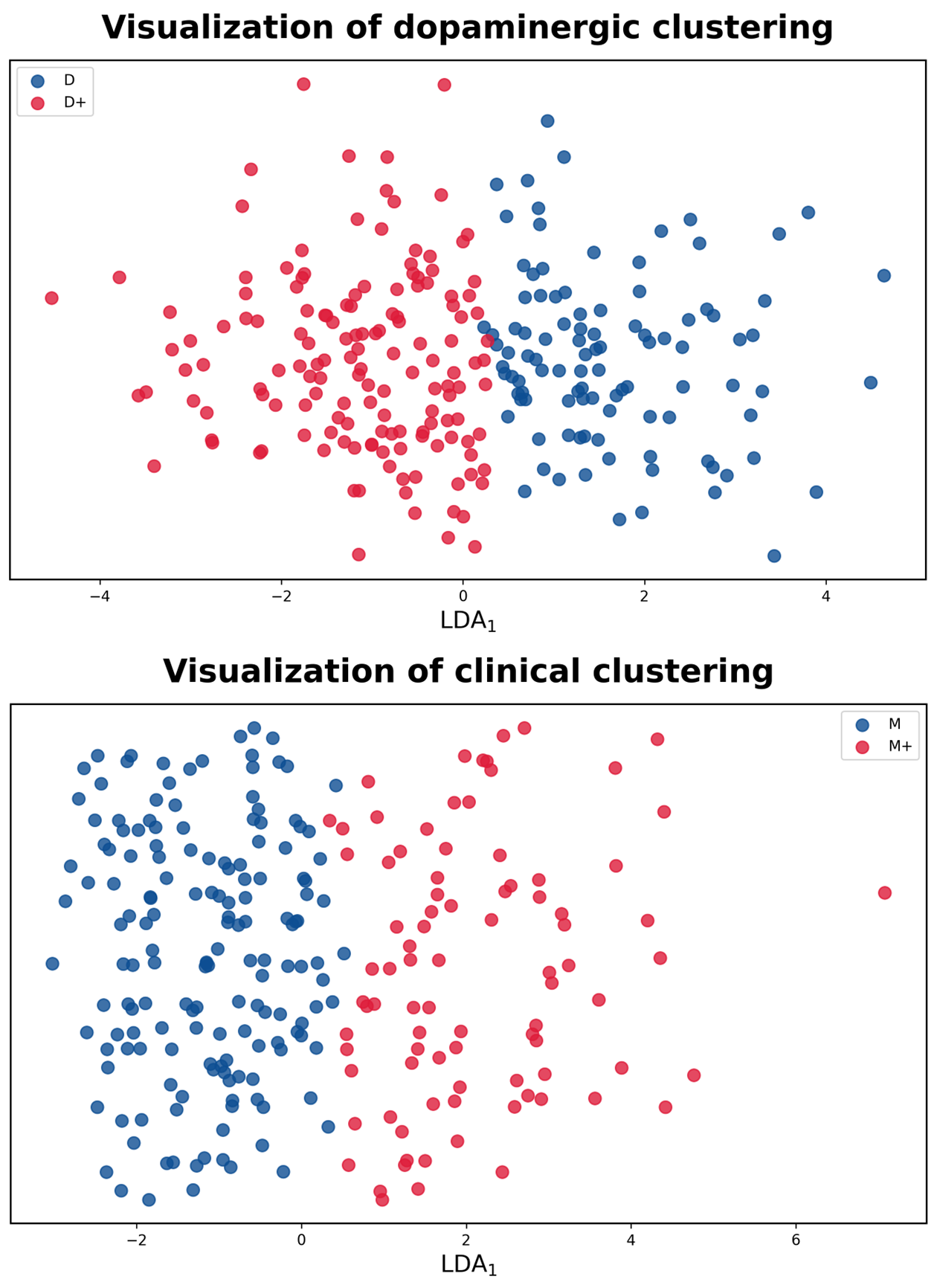

Supplement: Supplementary file 1 — Figure S1: Visualization of group separation using Linear Discriminant Analysis (LDA). [file ACN3-13-1398-s001.tif]
