# Supplementary material for: Clustering Algorithm Reveals Dopamine‐Motor Mismatch in Cognitively Preserved Parkinson's Disease
Source: Ann Clin Transl Neurol. 2026 Jan 28;13(7):1398–411. doi: 10.1002/acn3.70317 (PMC13358561; doi:10.1002/acn3.70317)
Supplement: Supplementary file 2 — Appendix S1: acn370317‐sup‐0002‐Appendix.docx. [file ACN3-13-1398-s002.docx]

**Clustering Algorithm Reveals Dopamine-Motor Mismatch in Cognitively Preserved Parkinson’s Disease**

**Running head: Dopamine-Motor Mismatch in PD**

Rachele Malito, MSc; Chiara Meneghini et al.

**Supplementary methods**

***Sensitivity analysis*** - We performed proportion tests, Chi-square tests and one-way ANOVA to compare demographic characteristics between the independent cohorts (PPMI cohort vs external cohort). Subsequently, a two-step cluster analysis was performed independently on the external cohort to validate the findings from the PPMI patient group (**Figure 1**).

The performed clustering analysis mimics the procedure adopted for the PPMI cohort, thus considering first the *DAT-SPECT* SBR values of left and right caudate and putamen and total MDS-UPDRS III score, as well as motor sub-scores.

Chi-square and non-parametric Kruskal-Wallis tests were performed to compare characteristics within the external cohort. Significance threshold set at 0.05.

A mixed linear model was employed to estimate the rate of longitudinal change in LEDD and MDS-UPDRS part III across the external subgroups, adjusting for age, sex and education.

**Supplementary results**

***Sensitivity analysis*** - The two independent cohorts analyzed were comparable in terms of numerosity, baseline age and sex.

*Baseline features****-*** Within the external cohort, the D cluster and D/M subgroup showed greater male prevalence (**Supplementary Table 12**, **Table 4**).

At baseline, D+/M+ demonstrated a higher prevalence of bradykinetic symptoms and a lower incidence of tremor, whereas D/M+ showed greater predominance of rigidity-related symptoms (**Table 4**).

*Longitudinal assessment* - The estimated annual rate of change in the total MDS-UPDRS Part III score was found to be significantly higher in D+/M+ (14.86.2 points) and D+ (5.65 points) (**Supplementary Table 12**, **Table 4**).

**Supplementary Figures**

**Supplementary Figure 1** - Visualization of group separation using Linear Discriminant Analysis (LDA)

Subjects are projected onto the first linear discriminant (LDA_1_), computed from either dopaminergic or clinical features. Data points are colored based on cluster membership (D+/D or M+/M), as previously defined through unsupervised clustering. LDA was used exclusively for dimensionality reduction and visualization; it did not influence or alter the original cluster assignments.

**Supplementary Tables**

**Supplementary Table 1 -** Cluster-wise silhouette statistics for D and M analyses in the PPMI and external cohorts.

| Cohort | Analysis | Cluster | N | Silhouette mean (SD) | % negative |
| --- | --- | --- | --- | --- | --- |
| PPMI | D | 0 | 102 | 0.8 (0.06) | 0.0 |
|  |  | 1 | 147 | 0.9 (0.07) | 0.0 |
|  | M | 0 | 161 | 0.6 (0.05) | 0.0 |
|  |  | 1 | 88 | 0.5 (0.12) | 0.0 |
| External Cohort | D | 0 | 64 | 0.7 (0.11) | 0.0 |
|  |  | 1 | 20 | 0.9 (0.06) | 0.0 |
|  | M | 0 | 39 | 0.6 (0.05) | 0.0 |
|  |  | 1 | 45 | 0.4 (0.13) | 0.0 |

Mean silhouette values (±SD) and the percentage of negative silhouettes are reported for each cluster, computed using Python (scikit-learn, silhouette_samples) applied to the Two step cluster assignments.

**Supplementary Table 2 –** Summary of clustering analysis of the PPMI cohort

|  | **D+** | **D** | **p-value** |
| --- | --- | --- | --- |
| N (%) | 147 (59%) | 102 (41.0%) | - |
| ***Brain imaging*** | | | |
| Cautate | 0.88 (0.2) | 1.27 (0.2) | **< 0.001 §** |
| *Caudate Left* | 0.88 (0.2) | 1.30 (0.2) | **< 0.001 §** |
| *Caudate Right* | 0.87 (0.2) | 1.25 (0.2) | **< 0.001 §** |
| Putamen | 1.14 (0.2) | 1.62 (0.2) | **< 0.001 §** |
| *Putamen Left* | 1.17 (0.2) | 1.65 (0.2) | **< 0.001 §** |
| *Putamen Right* | 1.12 (0.2) | 1.57 (0.3) | **< 0.001 §** |
| Ratio c/p | 0.76 (0.1) | 0.79 (0.1) | 0.225 |
| AI | 5.7 (3.9) | 6.0 (3.4) | 0.430 |
| Delay between first clinical evaluation and SPECT acquisition (months) | 1.2 (1.4) | 1.1 (1.0) | 0.404 |
|  | | | |
|  | **M+** | **M** | **p-value** |
| N (%) | 88 (35.3%) | 161 (64.7%) | - |
| ***Clinical assessment*** | | | |
| MDS-UPDRS III | 31.1 (6.8) | 15.8 (4.7) | **< 0.001 §** |
| Severity of motor signs (MDS-UPDRS-III) | | | |
| *Bradykinesia (tot. score range: 0-56)* | 17.1 (4.6) | 7.5 (3.0) | **< 0.001 §** |
| *Rigidity (tot. score range: 0-20)* | 6.3 (2.5) | 2.5 (1.2) | **< 0.001 §** |
| *Resting tremor (tot. score range: 0-40)* | 5.0 (3.6) | 4.4 (2.9) | 0.110 |
| *Postural (tot. score range: 0-24)* | 3.1 (2.0) | 1.5 (1.2) | **< 0.001 §** |

Linear regression models were performed to compare brain imaging and clinical features between clusters (D+/D and M+/M).

Abbreviations: Asymmetry Index (AI); Left (L); Movement Disorders Society - Unified Parkinson’s Disease Rating Scale (MDS-UPDRS); Number of participants or observations in a study or subgroup (N); Ratio Caudate/Putamen (c/p); Right (R); Single Photon Emission Computerized Tomography (SPECT).

Significant p-values (p<0.05) are reported in bold.

**Supplementary Table 3** - Comparison of brain imaging and motor impairment among the four subgroups of the PPMI cohort

|  | **D+/M+** | **D/M+** | **D+/M** | **D/M** | **p-value** | | | | | |
| --- | --- | --- | --- | --- | --- | --- | --- | --- | --- | --- |
|  |  |  |  |  | D+/M+ vs D/M+ | D+/M+ vs D+/M | D+/M+ vs D/M | D/M+ vs D+/M | D/M+ vs D/M | D+/M vs D/M |
| N (%) | 50 (20.1%) | 38 (15.3%) | 97 (39%) | 64 (25.7%) | - | - | - | - | - | - |
| ***Brain imaging*** | | | | | | | | | | |
| Caudate | 0.87 (0.2) | 1.27 (0.2) | 0.88 (0.2) | 1.28 (0.2) | **<0.001 §** | 0.648 | **<0.001 §** | **<0.001 §** | 0.719 | **<0.001 §** |
| *Caudate Left* | 0.89 (0.2) | 1.29 (0.2) | 0.88 (0.2) | 1.31 (0.2) | **<0.001 §** | 0.667 | **<0.001 §** | **<0.001 §** | 0.667 | **<0.001 §** |
| *Caudate Right* | 0.84 (0.2) | 1.25 (0.2) | 0.88 (0.2) | 1.26 (0.2) | **<0.001 §** | 0.183 | **<0.001 §** | **<0.001 §** | 0.838 | **<0.001 §** |
| Putamen | 1.15 (0.2) | 1.60 (0.2) | 1.14 (0.2) | 1.62 (0.2) | **<0.001 §** | 0.855 | **<0.001 §** | **<0.001 §** | 0.664 | **<0.001 §** |
| *Putamen Left* | 1.21 (0.2) | 1.63 (0.3) | 1.14 (0.2) | 1.66 (0.3) | **<0.001 §** | 0.108 | **<0.001 §** | **<0.001 §** | 0.621 | **<0.001 §** |
| *Putamen Right* | 1.10 (0.2) | 1.57 (0.3) | 1.14 (0.2) | 1.58 (0.2) | **<0.001 §** | 0.196 | **<0.001 §** | **<0.001 §** | 0.845 | **<0.001 §** |
| Ratio c/p | 0.75 (02) | 0.80 (0.1) | 0.77 (0.1) | 0.78 (0.09) | 0.138 | 0.461 | 0.212 | 0.241 | 0.470 | 0.510 |
| AI | 5.4 (3.7) | 5.9 (3.3) | 5.8 (4.0) | 6.1 (3.5) | 0.486 | 0.550 | 0.294 | 0.866 | 0.795 | 0.618 |
| Delay between first clinical evaluation and SPECT acquisition (months) | 1.1 (1.0) | 0.94 (0.8) | 1.3 (1.6) | 1.1 (1.1) | 0.497 | 0.453 | 0.851 | 0.229 | 0.391 | 0.475 |
| ***Clinical assessment*** | | | | | | | | | | |
| MDS-UPDRS III | 31.1 (6.8) | 31.1 (6.8) | 15.8 (4.5) | 15.9 (5.1) | 0.986 | **<0.001 §** | **<0.001 §** | **<0.001 §** | **<0.001 §** | 0.851 |
| Severity of motor signs (MDS-UPDRS-III) | | | | | | | | | | |
| *Bradykinesia (tot. score range: 0-56)* | 17.3 (5.0) | 16.8 (4.0) | 7.7 (3.0) | 7.2 (3.1) | 0.574 | **<0.001 §** | **<0.001 §** | **<0.001 §** | **<0.001 §** | 0.341 |
| *Rigidity (tot. score range: 0-20)* | 5.9 (2.7) | 6.8 (2.2) | 2.4 (1.5) | 2.6 (1.7) | 0.070 | **<0.001 §** | **<0.001 §** | **<0.001 §** | **<0.001 §** | 0.541 |
| *Resting tremor (tot. score range: 0-40)* | 5.0 (3.3) | 5.1 (3.9) | 4.1 (2.9) | 4.7 (2.9) | 0.824 | 0.118 | 0.680 | 0.104 | 0.543 | 0.209 |
| *Postural (tot. score range: 0-24)* | 3.3 (2.0) | 2.7 (1.9) | 1.6 (1.2) | 1.4 (1.2) | 0.156 | **<0.001 §** | **<0.001 §** | **<0.001 §** | **<0.001 §** | 0.485 |
| Clinical subtypes (*) | | | | | **<0.001** | | | | | |
| *Mild Motor-Predominant* | 19 (38%) | 14 (36.8%) | 56 (57.7%) | 46 (71.9%) | - | - | - | - | - | - |
| *Intermediate* | 15 (30%) | 11 (28.9%) | 36 (37.1%) | 17 (26.6%) | - | - | - | - | - | - |
| *Diffuse Malignant* | 16 (32%) | 13 (34.2%) | 5 (5.2%) | 1 (1.6%) | - | - | - | - | - | - |

Linear and logistic regression models were performed to compare brain imaging and clinical features at baseline between subgroups. Analyses were adjusted for sex, age, and baseline disease duration.

Abbreviations: Asymmetry Index (AI); Left (L); Movement Disorders Society - Unified Parkinson’s Disease Rating Scale (MDS-UPDRS); Number of participants or observations in a study or subgroup (N); Ratio Caudate/Putamen (c/p); Right (R); Single Photon Emission Computerized Tomography (SPECT).

(*****) Indicates variables analyzed using the chi-square test.

Significant p-values (p<0.05) are reported in bold.

**Supplementary Table 4** - Comparison of baseline demographic between D+/D clusters of the PPMI cohort

|  | **D+** | **D** | **p-value** |
| --- | --- | --- | --- |
| N (%) | 147 (59%) | 102 (41%) | - |
| Age at baseline | 62.2 (9.0) | 64.1 (9.0) | 0.096 |
| Age at onset | 60.3 (9.3) | 61.9 (8.9) | 0.186 |
| Age DAT-SPECT | 62.6 (9.0) | 64.5 (9.0) | 0.106 |
| Age MRI | 62.6 (8.5) | 64.2 (9.1) | 0.298 |
| Age at the start of therapy (LEDD) | 68.1 (8.6) | 69.9 (9.2) | 0.105 |
| Sex (% of males) | 93 (63.3%) | 73 (71.6%) | 0.219 |
| Education (years) | 15.4 (2.8) | 15.8 (2.8) | 0.349 |
| Disease Duration (years) | 0.18 (0.5) | 0.20 (0.5) | 0.837 |
| LEDD at baseline | 0.0 (0.0) | 0.0 (0.0) | - |
| DBS | 1 (7%) | 2 (2%) | 0.316 |
| Time between first clinical evaluation and DBS (years) | 9.2 (2.6) | 8.6 (3.3) | 0.601 |
| ***Ethnicity*** | | | |
| Hispanic/Latin | 2 (1.4%) | 1 (1.0%) | 1.00 |
| White | 139 (94.6%) | 101 (99%) | 0.086 |
| ***Family history*** | | | |
| Positive family history (%) | 28 (19%) | 32 (31.4%) | **0.037** |
| Positive family history-first generation (%) | 15 (10.2%) | 15 (14.7%) | 0.381 |

Comparison of baseline demographics between clusters using Chi-Squared and One-Way ANOVA (significance level of 0.05).

Abbreviations: Deep Brain Stimulation (DBS); Dopamine Active Transporter (DAT); Levodopa equivalent daily dose (LEDD); Magnetic Resonance Imaging (MRI); Number of participants or observations in a study or subgroup (N); Single Photon Emission Computerized Tomography (SPECT).

Significant p-values (p<0.05) are reported in bold.

**Supplementary Table 5** – Comparison of the baseline clinical features between D+/D clusters of the PPMI cohort

|  | **D+** | **D** | **p-value** |
| --- | --- | --- | --- |
| N (%) | 147 (59%) | 102 (41.0%) | - |
| ***Clinical assessment*** | | | |
| MDS-UPDRS II | 6.0 (3.8) | 5.4 (3.9) | 0.134 |
| MDS-UPDRS III | 20.9 (9.0) | 21.6 (9.3) | 0.899 |
| Severity of motor signs (MDS-UPDRS-III) | | | |
| *Bradykinesia (tot. score range: 0-56)* | 21 (9.1) | 21.6 (9.3) | 0.865 |
| *Rigidity (tot. score range: 0-20)* | 3.6 (2.6) | 4.2 (2.8) | 0.164 |
| *Resting tremor (tot. score range: 0-40)* | 4.4 (3.0) | 4.9 (2.3) | 0.399 |
| *Postural (tot. score range: 0-24)* | 2.2 (1.7) | 1.9 (1.6) | 0.066 |
| Clinical subtypes (*) | | | 0.431 |
| *Mild Motor-Predominant* | 75 (51%) | 60 (58.8%) | - |
| *Intermediate* | 51 (34.7%) | 28 (27.5%) | - |
| *Diffuse Malignant* | 21 (14.3) | 14 (13.7%) | - |
| ***Non-motor symptoms and signs*** | | | |
| MDS-UPDRS I | 1.5 (1.9) | 1.1 (1.3) | 0.122 |
| *Hallucinations and psychosis* | 0.03 (0.2) | 0.03 (0.2) | 0.908 |
| *Apathy* | 0.2 (0.6) | 0.2 (0.5) | 0.614 |
| *Pain* | 0.8 (0.9) | 0.7 (0.8) | 0.469 |
| *Constipation* | 0.5 (0.7) | 0.3 (0.5) | **0.016** |
| *Fatigue* | 0.7 (0.8) | 0.6 (0.7) | 0.553 |
| SCOPA-AUT | 11 (6.5) | 9.6 (5.6) | **0.025** |
| *Orofacial* | 1.0 (1.2) | 1.0 (1.2) | 0.807 |
| *Constipation* | 1.3 (1.4) | 0.9 (1.1) | **0.002** |
| *Urinary* | 4.4 (3.1) | 3.9 (2.6) | 0.067 |
| *Cardiovascular* | 0.5 (0.7) | 0.4 (0.8) | 0.166 |
| *Pupillo-Motor* | 0.3 (0.6) | 0.4 (0.6) | 0.630 |
| *Thermoregulatory* | 1.2 (1.4) | 0.9 (1.2) | 0.257 |
| *Sexual* | 1.3 (1.7) | 1.2 (1.6) | 0.476 |
| RBDsq | 3.3 (2.9) | 3.1 (2.6) | 0.615 |
| RBD disorders (RBDsq ≥ 6) | 29 (19.7%) | 18 (17.4%) | 0.558 |
| QUIPcs | 3.6 (1.7) | 3.5 (1.8) | 0.524 |
| GDS | 5.3 (1.6) | 5.1 (1.2) | 0.392 |
| STAI S | 46.1 (8.2) | 48.3 (4.7) | **0.018** |
| STAI T | 46 (4.0) | 46.2 (3.7) | 0.539 |
| ***Cognitive assessment*** | |  |  |
| MoCA (at baseline) | 26.9 (2.7) | 27.0 (2.3) | 0.352 |
| MoCA Slope | -0.4 (0.9) | -0.2 (0.8) | **0.027** |
| BJLO | 13.0 (2.4) | 13.0 (2.7) | 0.966 |
| LNS | 11.6 (2.8) | 11.9 (2.6) | 0.266 |
| Semantic fluency | 50.1 (10.5) | 51.4 (8.6) | 0.269 |
| HVLT, immediate recall | 44.4 (10.9) | 47.3 (10.1) | **0.017** |
| HVLT, delayed recall | 43.3 (11.3) | 46.3 (10.7) | **0.015** |
| HVLT, retention | 46.3 (12) | 47.9 (11) | 0.176 |
| HVLT, recognition index | 42.7 (12.1) | 47.5 (10) | **<0.001 §** |
| SDM | 44.3 (8.6) | 46.2 (9.8) | 0.073 |

Linear and logistic regression (with the Benjamini-Hochberg post hoc correction) were performed to compare clinical and cognitive features at baseline between clusters. Analyses were adjusted for sex, age, and disease duration at baseline. The Benjamini-Hochberg procedure was applied separately for clinical and cognitive section. Variables that were used to generate the clusters were excluded from the set of tests considered in the multiple comparison correction.

Missing value: MDS-UPDRS II (n=1); SCOPA-AUT - RBDsq - STAI - GDS – QUIPcs (n=3); BJLO - LNS - Semantic fluency - HVLT - SDM (n=4).

Abbreviations: Benton Judgment of Line Orientation (BJLO); Geriatric Depression Scale (GDS); Hopkins Verbal Learning Test-Revised (HVLT); Letter-Number Sequencing (LNS); Montreal Cognitive Assessment (MoCA); Movement Disorders Society - Unified Parkinson’s Disease Rating Scale (MDS-UPDRS); Number of participants or observations in a study or subgroup (N); Questionnaire for Impulsive-Compulsive Disorders in Parkinson’s Disease (QUIPcs); REM Sleep Behavior Disorder Screening Questionnaire (RBDsq); Scales for Outcomes in PD Autonomic (SCOPA-AUT); State-Trait Anxiety Inventory-State (STAI-S); State-Trait Anxiety Inventory-Trait (STAI-T); Symbol Digit Modality (SDM).

(*****) Indicates variables analyzed using the chi-square test.

Significant p-values (p<0.05) are reported in bold.

§ Significance survived Benjamini-Hochberg’s correction.

**Supplementary Table 6** - Comparison of CSF biomarkers between D+/D clusters of the PPMI cohort

|  | **D+** | **D** | **p-value** |
| --- | --- | --- | --- |
| N (%) | 147 (59%) | 102 (41.0%) | - |
| SAA (*) | | | 0.919 |
| *Inconclusive* | 2 (1.4%) | 2 (2.1%) | - |
| *Positive* | 129 (92.8%) | 89 (92.7%) | - |
| *Negative* | 8 (5.8%) | 5 (5.2%) | - |
| α-syn | 1483.8 (700.9) | 1617.7 (716.8) | 0.206 |
| Aβ1-42 | 877.0 (421.2) | 977.3 (468.4) | 0.078 |
| p-Tau181 | 14.3 (4.8) | 15.7 (5.9) | 0.154 |
| t-Tau | 162.8 (54.9) | 180.4 (61.7) | **0.044** |
| NfL | 13.6 (8.2) | 13.4 (5.9) | 0.178 |
| p-Tau181/α-syn | 0.009 (0.002) | 0.01 (0.002) | 0.050 |
| t-Tau/α-syn | 0.112 (0.02) | 0.118 (0.03) | 0.079 |
| Aβ1-42/α-syn | 0.61 (0.2) | 0.64 (0.23) | 0.129 |
| p-Tau181/Aβ1-42 | 0.016 (0.006) | 0.018 (0.01) | 0.416 |
| t-Tau/Aβ1-42 | 0.19 (0.06) | 0.21 (0.1) | 0.400 |
| p-Tau181/t-Tau | 0.084 (0.008) | 0.085 (0.007) | 0.525 |

Linear and logistic regression (with the Benjamini-Hochberg post hoc correction) were performed to compared CSF biomarkers at baseline between clusters. Analyses were adjusted for sex, age, and disease duration at baseline.

Missing value: SAA (n=14); α-syn (n=11); t-Tau (n=17); p-Tau_181_ (n=30); Aβ_1-42_ (n=14); NfL (n=11).

Abbreviations: Amyloid-β_1-42_ (Aβ_1-42_); Asymmetry Index (AI); α-synuclein (α-syn); α-syn seeding aggregation activity (SAA); Neurofilament light Chain (NfL); Number of participants or observations in a study or subgroup (N); Phosphorylated Tau (p-Tau_181_); total Tau (t-Tau).

(*****) Indicates variables analyzed using the chi-square test.

Significant p-values (p<0.05) are reported in bold.

§ Significance survived Benjamini-Hochberg’s correction.

**Supplementary Table 7 -** Model Comparing Rate of Change in motor domains and motor complications between D+/D clusters of the PPMI cohort

|  | **D** | |
| --- | --- | --- |
| Reference: D+ | Estimate (95% CI) | p-value |
| ***Rate of Change in Motor Score*** | | |
| LEDD | 25.4 (-6.8 to 57.6) | 0.122 |
| MDS-UPDRS-III score | 0.07 (-2.2 to 2.3) | 0.948 |
| *Bradykinesia sub-score* | 0.54 (-0.9 to 1.9) | 0.461 |
| *Tremor sub-score* | -0.75 (-1.5 to 0.04) | 0.062 |
| *Rigidity sub-score* | -0.13 (-0.8 to 0.5) | 0.702 |
| *Postural sub-score* | 0.23 (-0.1 to 0.6) | 0.176 |

The longitudinal comparison between clusters was performed using a linear mixed-effects model, with age, sex, and years of education as covariates.

Abbreviations: Confidence Interval (CI); Movement Disorder Society – Unified Parkinson’s Disease Rating Scale (MDS-UPDRS); Total Hours with Dyskinesia (THWD); Total Hours OFF (THOFF).

Significant p-values (p<0.05) are reported in bold.

**Supplementary Table 8** - Comparison of baseline demographic and genetic characteristics among the four subgroups of Parkinson’s disease population of the PPMI cohort.

|  | **D+/M+** | **D/M+** | **D+/M** | **D/M** | **p-value** |
| --- | --- | --- | --- | --- | --- |
| N (%) | 50 (20.1%) | 38 (15.3%) | 97 (39%) | 64 (25.7%) | - |
| Age at baseline | 64.4 (8.7) | 65.2 (8.6) | 61.1 (9.0) | 63.5 (9.2) | 0.062 |
| Age at onset | 62.6 (9.2) | 62.5 (8.8) | 59.3 (9.3) | 61.6 (9.0) | 0.108 |
| Age DAT-SPECT | 64.6 (8.8) | 65.6 (8.6) | 61.6 (9.0) | 63.9 (9.2) | 0.069 |
| Age MRI | 65.6 (6.8) | 65.3 (9.1) | 61.6 (9.1) | 63.6 (9.2) | 0.110 |
| Age at the start of therapy (LEDD) | 69.0 (6.1) | 70.7 (8.3) | 67.6 (9.6) | 69.5 (9.6) | 0.276 |
| Sex (% of males) | 35 (70%) | 26 (68.4%) | 58 (59.8%) | 47 (73.4%) | 0.298 |
| Education (years) | 15.8 (2.9) | 15.4 (2.7) | 15.2 (2.7) | 16.0 (2.9) | 0.328 |
| Disease Duration (years) | 0.16 (0.4) | 0.28 (0.5) | 0.20 (0.5) | 0.20 (0.5) | 0.965 |
| LEDD at baseline | 0.00 (0.00) | 0.00 (0.00) | 0.00 (0.00) | 0.00 (0.00) | - |
| DBS | 7 (14%) | 1 (2.6%) | 13 (13.4%) | 7 (10.9%) | 0.575 |
| Time between first clinical evaluation and DBS (years) | 7.7 (2.8) | 6 | 7.5 (2.7) | 7.7 (3.5) | 0.954 |
| ***Ethnicity*** | | | | | |
| Hispanic/Latin | 1 (2%) | 0 (0%) | 1 (1%) | 1 (1.6%) | 1.00 |
| White | 49 (98%) | 38 (100%) | 90 (92.8%) | 63 (98.4%) | 0.157 |
| ***Family history*** | | | | | |
| Positive family history (%) | 10 (20%) | 10 (26.3%) | 18 (18.6%) | 22 (43.4%) | 0.117 |
| Positive family history-first generation (%) | 6 (12%) | 2 (5.3%) | 9 (9.3%) | 13 (20.3%) | 0.112 |

Comparison of baseline demographics between subgroups using Chi-Squared and One-Way ANOVA were used.

Abbreviations: Deep Brain Stimulation (DBS); Dopamine Active Transporter (DAT); Levodopa equivalent daily dose (LEDD); Magnetic Resonance Imaging (MRI); Number of participants or observations in a study or subgroup (N); Single Photon Emission Computerized Tomography (SPECT).

Significant p-values (p<0.05) are reported in bold.

**Supplementary Table 9** - Models Comparing Rate of Change in motor domains and motor complications among subgroups of PD patients of the PPMI cohort

|  | **D+/M+** | | **D/M+** | | **D+/M** | | **D/M** | |
| --- | --- | --- | --- | --- | --- | --- | --- | --- |
| Reference: Group | Estimate (95% CI) | p-value | Estimate (95% CI) | p-value | Estimate (95% CI) | p-value | Estimate (95% CI) | p-value |
| ***Rate of Change in Motor Score*** | | | | | | | | |
| LEDD | 48.6 (9.57 to 87.6) | **0.015** | 16.4 (-28.3 to 61.2) | 0.471 | -5.09 (-35.4 to 25.2) | 0.742 | -37.2 (-71.9 to -2.51) | **0.036** |
| MDS-UPDRS-III score | 9.79 (7.3 to 12.3) | **<0.001** | 9.93 (7.1 to 12.8) | **<0.001** | -6.49 (-8.6 to -4.4) | **<0.001** | -6.42 (-8.8 to -4.1) | **<0.001** |
| *Bradykinesia sub-score* | 7.03 (5.5 to 8.5) | **<0.001** | 5.58 (3.7 to 7.4) | **<0.001** | -4.17 (-5.5 to -2.8) | **<0.001** | -4.24 (-5.7 to -2.7) | **<0.001** |
| *Tremor sub-score* | -0.46 (-1.4 to 0.5) | 0.353 | 0.79 (-0.3 to 1.9) | 0.158 | -0.45 (-1.2 to 0.3) | 0.267 | 0.43 (-0.4 to 1.3) | 0.337 |
| *Rigidity sub-score* | 2.07 (1.3 to 2.8) | **<0.001** | 2.89 (2.0 to 3.7) | **<0.001** | -1.52 (-2.2 to -0.9) | **<0.001** | -1.70 (-2.4 to 1.0) | **<0.001** |
| *Postural sub-score* | 0.91 (0.5 to 1.3) | **<0.001** | 0.62 (0.1 to 1.1) | **0.009** | -0.37 (-0.7 to -0.03) | **0.032** | -0.68 (-1.0 to -0.3) | **<0.001** |

The longitudinal comparison between subgroups was performed using a linear mixed-effects model, with age, sex, and years of education as covariates.

Abbreviations: Confidence Interval (CI); Movement Disorder Society – Unified Parkinson’s Disease Rating Scale (MDS-UPDRS); Total Hours with Dyskinesia (THWD); Total Hours OFF (THOFF)

Significant p-values (p<0.05) are reported in bold.

**Supplementary Table 10 -** Models comparing the rate of change in MDS-UPDRS III score and motor domains conducted pair-to-pair comparisons between subgroups of the PPMI cohort.

|  | **D/M+** | | **D+/M** | | **D/M** | |
| --- | --- | --- | --- | --- | --- | --- |
| Reference group: D+/M+ | Estimate (95% CI) | p-value | Estimate (95% CI) | p-value | Estimate (95% CI) | p-value |
| ***Rate of Change in Motor Score*** | | | | | | |
| MDS-UPDRS-III score | 0.63 (-2.2 to 3.4) | 0.659 | -11.78 (-14.0 to -9.5) | **<0.001** | -12.47 (-14.9 to -10.1) | **<0.001** |
| *Bradykinesia sub-score* | -0.87 (-2.6 to 0.9) | 0.328 | -8.15 (-9.5 to -6.8) | **<0.001** | -8.69 (-10.2 to -7.2) | **<0.001** |
| *Tremor sub-score* | 1.04 (-0.3 to 2.4) | 0.128 | 0.09 (-1.0 to 1.2) | 0.867 | 0.68 (-0.5 to 1.8) | 0.245 |
| *Rigidity sub-score* | 0.81 (-0.1 to -1.7) | 0.086 | -2.57 (-3.3 to -1.8) | **<0.001** | -2.89 (-3.7 to -2.1) | **<0.001** |
| *Postural sub-score* | -0.20 (-0.7 to 0.3) | 0.468 | -0.95 (-1.4 to -0.5) | **<0.001** | -1.22 (-1.7 to -0.8) | **<0.001** |
|  | | | | | | |
|  | **D+/M+** | | **D+/M** | | **D/M** | |
| Reference group: D/M+ | Estimate (95% CI) | p-value | Estimate (95% CI) | p-value | Estimate (95% CI) | p-value |
| ***Rate of Change in Motor Score*** | | | | | | |
| MDS-UPDRS-III score | -0.63 (-3.4 to 2.2) | 0.659 | -12.41 (-14.9 to -9.9) | **<0.001** | -13.1 (-15.7 to -10.5) | **<0.001** |
| *Bradykinesia sub-score* | 0.87 (-0.9 to 2.6) | 0.328 | -7.28 (-8.8 to -5.7) | **<0.001** | -7.82 (-9.50 to -6.2) | **<0.001** |
| *Tremor sub-score* | -1.04 (-2.4 to 0.3) | 0.127 | -0.95 (-2.1 to 0.2) | 0.117 | -0.36 (-1.6 to 0.9) | 0.577 |
| *Rigidity sub-score* | -0.81 (-1.7 to 0.1) | 0.086 | -3.38 (-4.2 to -2.6) | **<0.001** | -3.70 (-4.6 to 2.8) | **<0.001** |
| *Postural sub-score* | 0.20 (-0.3 to 0.7) | 0.468 | -0.75 (-1.2 to -0.3) | **0.002** | -1.02 (-1.5 to -0.5) | **<0.001** |

The comparison of the rate of change in motor domains between subgroups was performed using pair-to-pair comparisons within a linear mixed-effects model, with age, sex, and years of education as covariates.

Abbreviations: Confidence Interval (CI); Movement Disorder Society - Unified Parkinson’s Disease Rating Scale (MDS-UPDRS).

Significant p-values (p<0.05) are reported in bold.

**Supplementary Table 11** – Direct and indirect associations of D+/M+ with MoCA slope mediated via CSF biomarkers

|  |  | β | 95% CI lower | 95% CI upper | p-value |
| --- | --- | --- | --- | --- | --- |
| α-syn | ACME | -0.015 | -0.06 | 0.01 | 0.358 |
|  | ADE | -0.414 | -0.84 | -0.08 | **0.010** |
|  | Total effect | -0.429 | -0.84 | -0.10 | **0.008** |
|  | PM (%) | 3.465 | -1.00 | 77.34 | 0.362 |
| Aβ1-42 | ACME | -0.050 | -0.11 | -0.01 | **0.008** |
|  | ADE | -0.334 | -0.70 | -0.03 | **0.020** |
|  | Total effect | -0.384 | -0.74 | -0.09 | **0.004** |
|  | PM (%) | 12.998 | 6.60 | 112.82 | **0.012** |
| t-Tau | ACME | -0.006 | -0.06 | 0.02 | 0.734 |
|  | ADE | -0.426 | -0.82 | -0.11 | **0.010** |
|  | Total effect | -0.432 | -0.84 | -0.14 | **0.004** |
|  | PM (%) | 1.409 | -2.60 | 61.00 | 0.738 |
| p-Tau181 | ACME | 0.007 | -0.02 | 0.05 | 0.772 |
|  | ADE | -0.474 | -0.86 | -0.14 | **0.000** |
|  | Total effect | -0.468 | -0.86 | -0.15 | **0.000** |
|  | PM (%) | 1.401 | -14.28 | 6.91 | 0.772 |
| NfL | ACME | 0.002 | -0.01 | 0.06 | 0.894 |
|  | ADE | -0.447 | -0.85 | -0.14 | **0.004** |
|  | Total effect | -0.445 | -0.82 | -0.14 | **0.004** |
|  | PM (%) | 0.433 | -4.88 | 3.03 | 0.890 |
| Aβ1-42 /α-syn | ACME | -0.025 | -0.07 | 0.001 | 0.098 |
|  | ADE | -0.383 | -0.78 | -0.10 | **0.004** |
|  | Total effect | -0.408 | -0.80 | -0.13 | **0.004** |
|  | PM (%) | 6.164 | 0.85 | 50.95 | 0.098 |
| t-Tau/α-syn | ACME | -0.010 | -0.05 | 0.01 | 0.488 |
|  | ADE | -0.441 | -0.85 | -0.14 | **0.004** |
|  | Total effect | -0.452 | -0.86 | -0.15 | **0.002** |
|  | PM (%) | 2.312 | -16.15 | 5.20 | 0.490 |
| p-Tau181/α-syn | ACME | 0.003 | -0.05 | 0.05 | 0.932 |
|  | ADE | -0.493 | -0.92 | -0.18 | **0.000** |
|  | Total effect | -0.490 | -0.93 | -0.17 | **0.002** |
|  | PM (%) | 0.591 | -28.63 | 5.49 | 0.934 |
| t-Tau/Aβ1-42 | ACME | -0.040 | -0.13 | 0.001 | 0.104 |
|  | ADE | -0.366 | -0.74 | -0.07 | **0.026** |
|  | Total effect | -0.406 | -0.79 | -0.11 | **0.012** |
|  | PM (%) | 9.892 | 1.49 | 113.54 | 0.116 |
| p-Tau181/ Aβ1-42 | ACME | -0.035 | -0.16 | 0.01 | 0.232 |
|  | ADE | -0.406 | -0.88 | -0.10 | **0.008** |
|  | Total effect | -0.441 | -0.88 | -0.11 | **0.006** |
|  | PM (%) | 7.936 | -3.45 | 55.50 | 0.234 |
| p-Tau181/t-Tau | ACME | 0.006 | -0.03 | 0.05 | 0.758 |
|  | ADE | -0.474 | -0.89 | -0.16 | **0.004** |
|  | Total effect | -0.468 | -0.89 | -0.15 | **0.006** |
|  | PM (%) | 1.233 | -28.71 | 4.18 | 0.764 |

Abbreviations: Amyloid-β_1-42_ (Aβ_1-42_); α-synuclein (α-syn); average causal mediation effect (ACME); average direct effect (ADE); confidence interval (CI); Neurofilament light Chain (NfL); Phosphorylated Tau (p-Tau_181_); proportion mediated (PM); total Tau (t-Tau).

Significant p-values (p<0.05) are reported in bold.

**Supplementary Table 12** - Comparison of the baseline clinical features and longitudinal rate of change in LEDD, MDS-UPDRS III Score between D+/D clusters of the external cohort

|  | **D+** | **D** | **p-value** | |
| --- | --- | --- | --- | --- |
| N (%) | 20 (23.8%) | 64 (76.2%) | - | |
| Age at baseline | 66 (8.7) | 64.1 (9.7) | 0.434 | |
| Age at onset | 65 88.8) | 63.4 (9.7) | 0.514 | |
| Sex (% of males) | 7 (35%) | 45 (70.3%) | **0.010** | |
| Education (years) | 9.4 (4.4) | 9.1 (3.9) | 0.775 | |
| Disease Duration (years) | 1.4 (0.5) | 1.3 (0.5) | 0.322 | |
| ***Clinical assessment at baseline*** | | | | |
| MDS-UPDRS-III | 15.2 (14.8) | 11.8 (7.0) | 0.164 | |
| *Bradykinesia* | 7 (46.7%) | 24 (43.6%) | 1.00 | |
| *Rigidity* | 3 (20%) | 22 (40%) | 0.259 | |
| *Resting tremor* | 11 (73.3%) | 39 (70.9%) | 1.00 | |
| *RBD* | 3 (42.7%) | 10 (50%) | 1.00 | |
| MMSE | 27.7 (2.8) | 27.9 (1.6) | 0.768 | |
| ***Clinical trajectories at follow-up*** | | | | |
| **Rate of Change in Motor Score** | | | | |
|  | LEDD | | MDS-UPDRS-III score | |
| Reference: D+ | Estimate (95% CI) | p-value | Estimate (95% CI) | p-value |
| D | -8.38 (-46.6 to 29.8) | 0.667 | 5.65 (0.44 to 10.9) | **0.033** |

Chi-square tests and one-way ANOVA were used to compare baseline demographic and clinical data between clusters within the external cohort. Longitudinal comparisons between groups within the external cohort were performed using a linear mixed-effects model.

Abbreviations: Levodopa equivalent daily dose (LEDD); Mini-Mental State Examination (MMSE); Movement Disorders Society - Unified Parkinson’s Disease Rating Scale (MDS-UPDRS); Number of participants or observations in a study or subgroup (N); REM sleep Behaviour Disorder (RBD).

Significant p-values (p<0.05) are reported in bold.
